# Using co-production to implement patient reported outcome measures in third sector organisations: a mixed methods study

**DOI:** 10.1186/s41687-022-00485-4

**Published:** 2022-07-19

**Authors:** Alexis Foster, Alicia O’Cathain, Janet Harris, Guy Weston, Lucy Andrews, Olga Andreeva

**Affiliations:** 1 University of Sheffield, Sheffield, UK; 2 SOAR Community Organisation, Sheffield, UK; 3 Manor and Castle Development Trust, Sheffield, UK; 4 HSE University and Federal Research Institute for Health Organization and Informatics of Ministry of Health of the Russian Federation, Moscow, Russia

**Keywords:** Patient-reported outcomes, Outcome assessment, Implementing, Third sector organisations

## Abstract

**Background:**

Third sector organisations such as charities and community groups are using Patient Reported Outcome Measures (PROMs) at an aggregated service level to demonstrate their impact to commissioners to generate or retain funding. Despite this motivation, organisations can struggle with implementing PROMs. Previous studies have identified facilitators including organisations using an appropriate measure, co-producing the PROMs process with staff, and investing resources to support the use of measures. However, to date no studies have applied this learning to third sector organisations to evaluate whether taking an evidence-informed implementation approach improves the use of PROMs.

**Methods:**

A Community-Based Participatory Research approach was used which involved university-based researchers supporting two third sector organisations to implement PROMs. The researchers provided evidence-informed advice and training. The organisations were responsible for implementing PROMs. The researchers evaluated implementation through a mixed methods approach including five key informant interviews, four evaluation groups and analysis of collected PROMs data (n = 313).

**Results:**

Both third sector organisations faced considerable constraints in incorporating known facilitators and addressing barriers. The organisations involved staff in choosing an acceptable measure. However, competing priorities including external pressures to use specific PROMs, busy workloads and staff opinions created challenges to using measures. Investment of time and energy into developing an outcomes-based organisational culture was key to enable the prioritisation of PROMs. For example, discussing PROMs in supervision so that they were viewed as part of people’s job roles. Organisations found that implementation took several years and was disrupted by other pressures.

**Conclusions:**

Whilst organisations were motivated to implement PROMs to obtain or retain funding, they faced considerable practical and ideological challenges. Consequently, some stakeholders felt that alternative methods to measuring impact could potentially be more feasible than PROMs.

**Supplementary Information:**

The online version contains supplementary material available at 10.1186/s41687-022-00485-4.

## Plain English summary

Organisations may use Patient Reported Outcome Measures (PROMs). Service-users complete a set of questions about their health before and after having help to see if their health has improved. However, staff find it hard to use PROMs. To solve this, researchers worked with two charities to help them use PROMs. Together, we chose a PROM and trained staff. We found that managers had to spend a lot of time trying to get PROMs used. This took several years and cost money. Staff needed a lot of support to use PROMs. Given the challenges, organisations may find it easier to use other types of methods to test if their service works.

## Introduction

Organisations delivering health and wellbeing services often use Patient Reported Outcome Measures (PROMs) to measure their impact on people’s health and wellbeing [1]. PROMs are standardised questionnaires that enable a person to rate their health, wellbeing or symptoms [2]. Measures include the Warwick-Edinburgh Mental Wellbeing Scale (WEMWBS) [3] EQ-5D-5L [4] and the Adult Social Care Outcomes Toolkit (ASCOT) [5]. Changes in someone’s health can be measured by completing the PROM at more than one time point, for example before and after attending a service. Organisations may aggregate the results from a number of service-users to evaluate the impact of a service [6]. For example, NHS England has recommended the use of PROMs in social prescribing programmes [7]. In the United States of America, the PROMIS programme is developing item-banks of measures for organisations to use [8].

Despite being motivated to use PROMs to demonstrate impact and secure funding [9], organisations have often struggled with implementation, characterised by low completion rates of measures [10]. Implementation is the process between an organisation deciding to adopt PROMs and their use within the organisation [11]. A number of contextual and process related factors impacting on the implementation of PROMs have been identified [9]. They may be a facilitator or barrier depending on their execution [12]. Identified contextual factors include organisations having to implement PROMs because external commissioners demand their use in return for funding [9]. Internally, organisations need a strategic commitment to using PROMs and resource the collection and analysis of measures e.g. investing in data management systems [13–15]. Having an Implementation Lead responsible for getting measures used [16] and consulting and training front-line workers appears beneficial [17, 18].

One type of organisation facing challenges implementing PROMs are Third Sector Organisations (TSOs). TSOs, also known as charities and community groups, are increasingly commissioned to deliver health and wellbeing services [12]. For example, within the United Kingdom, TSOs are viewed as a key provider within health care [19]. TSOs deliver a range of services including social groups for people experiencing bereavement, healthy eating courses, social prescribing and mental wellbeing programmes. TSOs are non-profit organisations which exist to bring change to the lives of their targeted population and are separate to the state [20, 21]. TSOs are often commissioned or funded on short-term contracts by government or grant giving agencies and are required to demonstrate their impact through PROMs to justify their funding [22]. Despite this motivation, TSOs, like clinical services with a long history of using PROMs, can struggle with implementation [23]. TSOs report that having to use inappropriate measures mandated by commissioners is a key barrier [9]. Consequently, the use of PROMs may be improved by organisations choosing their measure and taking an evidence-informed approach to implementation. This approach has been successful in clinical services including oncology [18] and rehabilitation [13]. The aim of the study was to evaluate the experiences of two TSOs taking an evidence-informed PROMs implementation approach.

## Methods

### Community-based participatory research

We used Community-Based Participatory Research (CBPR), which involved university-based researchers supporting two TSOs (Organisation A and B) with both implementing PROMs and evaluating their experiences. We used CBPR because taking a collaborative approach can facilitate the implementation of PROMs [24]. CBPR is a widely used type of participatory action research [25], where researchers and stakeholders are partners in the research process, co-producing knowledge together that is beneficial for all [26, 27]. The design of CBPR projects, role of different partners and levels of participation between researchers and other stakeholders varies between studies but there is the underlying principle of partnership and having an impact [27].

**Fig. 1:**
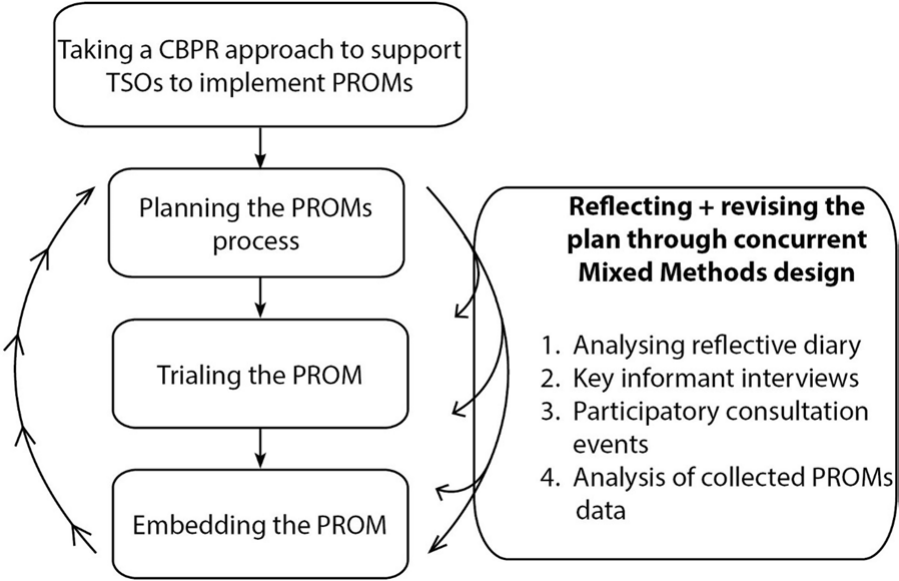
Diagram documenting the Community-Based Participatory Research approach

We used an action research spiral model [28], which involved the organisations making decisions on, and undertaking the implementation of PROMs. The lead author (AF) supported this process by sharing their knowledge on implementing PROMs and providing training to front-line workers on collecting measures. Alongside, researchers evaluated the organisations’ experiences of implementation, feeding back learning so that TSOs could improve the process (Fig. 1). The TSO leaders felt that the researchers were best placed to undertake the evaluation because they had the time and technical ability.

The collaboration between the researchers and the TSOs meant that together we were co-constructing the implementation process and learning [29]. This contrasts with other study designs, where stakeholders are solely participants [30]. These dual roles can create tensions including decisions on the PROMs implementation process and prioritising research activities. To manage these tensions, the lead author recorded reflections in her diary and had regular meetings with TSO stakeholders and an experienced participatory researcher (JH).

### Description of the TSOs involved

We involved two organisations that were at different stages of their implementation journey, enabling us to learn more about the process. Both organisations delivered a range of support including employment and advocacy services in neighbourhoods experiencing socioeconomic deprivation [31]. The TSOs were relatively large, each having an income of over £1,000,000 annually [32, 33] generated from a mixture of commissioned contracts with statutory agencies, funding from grant-giving organisations and income generating activities. Approximately 3000 service-users were supported in each organisation per year. Each TSO had a health and wellbeing team consisting of approximately 10 front-line workers who delivered a range of support including individual lifestyle coaching, social prescribing and men’s groups [34]. The intention was for PROMs to be utilised by all workers in these teams.

The inclusion of the organisations was opportunistic as the researchers had prior relationships with the TSOs and knew they were interested in improving the use of PROMs in their organisations. Both TSOs had struggled to use PROMs mandated by commissioners, partly because the organisations considered the previous measures unsuitable. This was because they felt the questions were difficult to understand or did not consider relevant constructs. The organisations needed to implement PROMs to satisfy their commissioners and hoped that the study would help them to use a measure within their organisation that was acceptable to staff.

The two TSOs were involved differently within the study because they were at different stages of implementation. This study ran formally between June 2018 and January 2020 (19 months). Organisation A became involved because they were at the start of their PROMs’ implementation process. During the study, the focus was on choosing a measure, deciding how to implement the PROM and attempting to utilise it. In contrast, we had been working with Organisation B since 2016 to choose a measure. During this study, the organisation sought to use the PROM routinely within their services.

### Implementation process

University-based researchers supported the two TSOs with implementation [35]. The work was led by AF, who was undertaking the study as part of their PhD. Two supervisors and a post-graduate student supported the study. The researchers shared learning from previous studies with the organisations, who then made their own decisions on designing how to collect, process and use PROMs data. In both organisations, a manager acted as the Implementation Lead, they were responsible for making decisions and progressing implementation.

The researchers met regularly with each Implementation Lead during the study to discuss implementation issues including which measures to use, designing a PROMs process and to identify solutions to arising issues. Alongside giving evidence-informed advice, the researchers provided additional capacity including writing guidelines for collecting measures and delivering training on using PROMs data to front-line workers. After designing the PROMs process, the two TSOs planned to trial, then embed, the PROMs by using the chosen measures throughout their wellbeing services.

### Evaluation

We used a mixed methods research design to evaluate the implementation of PROMs. We analysed collected PROMs data to explore completion rates and variation between staff members. We undertook several qualitative approaches including key informant interviews, evaluation meetings and analysed a researcher’s reflective diary to explore facilitators and barriers to implementation.

#### Qualitative methods and analysis

##### Evaluation meetings

We held evaluation meetings in each organisation. A researcher (AF) facilitated the meetings. They were attended by front-line workers, managers and Implementation Leads. The groups discussed implementation issues and potential solutions, which were then acted on by the organisations. At the beginning of the meetings, the researcher explained the study and informed attendees that notes would be taken of arising issues. People were able to leave the meeting if they did not want to be part of the study, this opt out consent process is commonly used within participatory research [36] and received ethical approval.

Three events were held in Organisation A, two were in Months 4 and 6 to discuss the choice of PROM. The third event was in Month 18 to reflect on progress. In Organisation B, one event was held in Month 16 to review the implementation process. Fewer events were held in Organisation B because they were at a later stage of their implementation journey. We used visual methods including brainstorming to capture the discussions, these were recorded within the researchers’ (AF’s) reflective diary [37].

##### Key informant interviews

Key informant interviews were undertaken in Months 15–18 to gain the insights of people pivotal to implementation [38]. The sample size was small because the interviews were supplementing other data sources. AF undertook the interviews, using a topic guide developed for each interviewee. Written consent was taken and each interview was audio-recorded [39]. These were transcribed verbatim, anonymised and uploaded to NVivo Version 12.

##### Reflective diary

As the lead researcher, AF kept a diary throughout the study to reflect on the implementation process (similar to the use of field notes in ethnography) [40]. After any contact with stakeholders, the researcher recorded notes about the encounters along with reflections. The diary was anonymised and uploaded to NVivo Version 12 for analysis, using the same process as for interview transcripts.

#### Qualitative data analysis

AF, supported by the other researchers, undertook thematic analysis to analyse the qualitative data collected through the methods described above [41]. Analysis entailed:

- *Familiarisation* We read the transcripts and the reflective diary several times.
- *Coding* We grouped findings by commonality.
- *Searching for themes* We identified connections between the codes to develop themes.
- *Reviewing the themes* We checked whether the themes reflected the data.
- *Defining and revising the themes* We developed the overarching message of each theme.

#### Analysis of routinely collected data

We used the collected PROMs data to analyse how extensively the measures were used in each organisation. Although our intention was to include both organisations, we could only analyse data for Organisation B because Organisation A had not reached a stage of using the measures extensively nor uploaded them into their data management system. Organisation B provided an anonymised dataset of PROMs scores. This consisted of a service-user reference number and information on each measure completed including the date of administration, scores for each question and name of the staff member who administered the PROM. The data was collected between January 2019 and February 2020. Organisation B did not link demographic or service use data to the PROMs data, so this was not available for analysis. AF converted the file into SPSS, cleaned the dataset and conducted descriptive analysis to explore how completion rates differed between specific front-line workers and over time. We hypothesised that completion rates would increase as PROMs became embedded within the organisation.

#### Integrating the findings

A ‘following the thread’ technique was used to synthesise the findings from the evaluation groups, key informant interviews, reflective diary and analysis of PROMs data [42]. This entailed identifying a finding in one data source and exploring how it transpired in the other data sources. For example, the quantitative data indicated that PROMs collection decreased over time and we used the qualitative data to identify reasons for the reduction. The findings below are reported in themes that draw on both the qualitative and quantitative data.

#### Reporting

The Mixed Methods Appraisal Tool was used as a reporting tool to ensure that relevant information was included in the manuscript [43] (Additional file 1).

#### Ethical approval

Approval was granted by the University of Sheffield (Application: 020700).

## Results

### The sample

#### Evaluation meetings

In Organisation A, a mixture of managers and front-line workers attended the meetings. Twelve people attended the first two meetings and 7 attended the final meeting. In Organisation B, 8 front-line workers attended the single meeting. The different make-up of groups reflected the preferences of the individual TSOs. Events lasted between 90 and 120 min and were held on the organisations' premises.

#### Key Informant Interviews

Five participants were interviewed, three worked at Organisation B (Implementation Lead, Senior Manager and a front-line worker) and two were from Organisation A (Implementation Lead and a front-line worker). The interviews generally lasted about 60 min, with three taking place at the organisations’ premises and two at the university.

#### Analysis of routinely collected PROMs data

The dataset from Organisation B consisted of 313 service-users who had completed a PROM at the start of attending a wellbeing activity, 62 of these completed a second PROMs after receiving support (19.8%).

### Overview of themes

Both TSOs designed a bespoke PROM because stakeholders felt that this would help workers to use the measure. However, organisations faced significant challenges including competing priorities and having to develop an outcomes-based organisational culture. Four key themes were: (1) Rationale for utilising PROMs. (2) Co-producing an appropriate bespoke measure. (3) Competing priorities and (4) Embedding PROMs into an organisation.

### Rationale for utilising PROMs

Both TSOs were implementing PROMs because there was an expectation from commissioners that the organisations needed to demonstrate outcomes to receive funding. The TSOs viewed PROMs as something they had to use on an aggregate level to demonstrate the impact of the service. A secondary purpose was front-line workers using the tool with service-users to reflect on their needs and progress. Previously, commissioners had mandated specific measures that the TSOs considered inappropriate for use within their organisation. In response, the organisations hoped that choosing a PROM themselves would result in staff being more engaged and commissioners no longer imposing their choice of measure.

So the experience of [a previous PROM] was so negative that actually, you’re not actually getting very good quality stuff because you’re just thinking, well you’re filling it in for the sake of it. [Implementation Lead A]

There were differences in how the two TSOs embraced PROMs. Organisation B proactively committed to becoming an outcomes-focused organisation that used PROMs. In contrast, the Implementation Lead in Organisation A did not feel that PROMs were a valid way to measure impact but felt obliged to use measures in response to the commissioning environment.

It’s not relevant and it’s not appropriate to ask customers but I do feel quite confident that the staff are embedded in the community and have got the links and the relationships with the people participating. [Implementation Lead A]

### Co-producing an appropriate bespoke measure

Both TSOs involved front-line workers in designing an organisation-specific bespoke measure. Neither set out to design a measure but felt existing measures did not meet their needs. This was because the front-line workers and Implementation Leads wanted a PROM which was short, used clear language and included graphics to help service-users with understanding the text. The organisations felt this was more of a priority than using a measure which had undergone psychometric testing. The organisations involved front-line workers in choosing a PROM because they hoped this would motivate them to use it. We hypothesised that the bespoke PROMs would be used by front-line workers because they considered it suitable for their service-users.

Spoke to front-line workers at Organisation B and they feel the design of the PROM was much better than what they had to use previously and reported that service-users find the graphics useful. [Extract from reflective diary]

The PROM tool that we developed, I’m really proud of that because it was developed using staff, yourselves and all that kind of stuff, so it was something that was co-produced, so it had meaning to all those stakeholder groups. [Senior Manager B]

Organisation B’s measure combined questions from several validated measures including the Short-version of WEMWBS and the ASCOT. However, Organisation B developed one question themselves, asking service-users ‘*How are you feeling’* (Additional file 2). They added this as an introductory question to orientate service-users. In contrast, Organisation A predominantly wrote their own questions because they did not feel that questions from existing PROMs were suitable for their service-users. The questions focused on service-users’ ability to cope with problems and development of support networks. For example, *How well do you cope with managing problems?* (Additional file 3). The measures did not undergo psychometric testing as the aim of the study was to understand whether the PROMs would be used. Despite having acceptable measures, the organisations faced challenges implementing them, these barriers are discussed below.

### Competing priorities

The organisations had to take a pragmatic approach to implementing PROMs because their staff were juggling multiple priorities including completing PROMs mandated by commissioners and busy workloads. However, prioritisation also appeared to be influenced by an individual’s personal opinions, with people who valued measures seemingly more likely to find time to progress implementation than people who felt that PROMs were inappropriate for use in TSOs.

Having met with [Name of front-line worker], I am really concerned about how we will get them using the PROM. They appear resistant to any changes being enforced. [Extract from reflective diary]

#### Prioritising commissioner mandated PROMs

The organisations faced an ongoing process of implementing different PROMs at the request of commissioners. These PROMs had to be prioritised over the organisation’ measures as funding was conditional on their use. TSOs had hoped that commissioners would accept their chosen measure so that they could take one organisational approach to collecting PROMs, but this did not happen. Implementation Lead B reflected that they may need to amend their bespoke measure to avoid front-line workers having to collect multiple PROMs.

We’ve been told we are to report these outcome measures […].so it could be rather than them [front-line workers] doing two questionnaires we might then make the decision to amend it. [Implementation Lead B]

#### Implementation Leads relying on support from researchers

The Implementation Leads faced multiple, time critical workload responsibilities including grant applications, managing staff and overseeing service delivery. Consequently, the Implementation Leads valued having support from researchers who had the time and skills to dedicate to implementation. Researchers provided a range of support including delivering training to front-line workers and designing methods of displaying the collected data. The organisations felt that this input was essential.

Not a chance we would have done that. We could have started it off, but we would never have finished it […] we can’t do it on our own. Really so undoubtedly and crucially we wouldn’t be able to do it without the university. [Implementation Lead B]

#### Front-line workers prioritising support for service-users

In both organisations, some front-line workers reported finding it difficult to complete PROM related tasks because they prioritised spending time supporting service-users. For example, people felt that they did not have time to input collected measures into data management systems and wanted administrative support for this task. This concern may have partly arisen because of the PROMs primarily being used to demonstrate service impact rather than as a progress tool with individual service-users. To address the time pressure, Implementation Leads made changes to the front-line workers’ roles so that they would have time to spend on PROMs related tasks.

I think that message is getting through that we need, what we need to do is quality and we need to be able to show and prove that its good and so and giving them [front-line workers] permission to take on less volume [of service-users] and giving them permission to have time in the office to do the paperwork and that, that is valid. [Implementation Lead A]

#### Having a flexible PROMs process to enable the prioritisation of service-user needs

The organisations designed a flexible PROMs data collection process because they wanted to prioritise the needs of service-users. Examples include being able to administer the PROMs in person or over the telephone and administering measures when it felt right for the service-user rather than at specific timepoints. However, front-line workers felt there was almost too much flexibility because they were not always sure when to utilise measures. This was detrimental for data quality.

So, it was retrospective because we weren’t using it when the group set up in January and I tried to get them to think where they were when they first came, where they are now and hopefully where they’re gonna be, well let’s just say just before Christmas, if they want to do it again. [Front-line worker A]

### Embedding PROMs into an organisation

The TSOs invested time into developing their culture including supporting front-line workers so that PROMs were embedded into the norms and practices of the organisation. However, the organisations struggled to sustain the use of their bespoke measure indicating that TSOs faced considerable implementation challenges especially when a PROM was not mandated by commissioners.

#### Developing organisational culture to be receptive to PROMs

Stakeholders found that they needed to invest time in developing their organisational culture to be compatible with outcome measurement. In Organisation B, the Implementation Lead and senior managers invested time in evolving the norms, values, and expectations of the organisation so that PROMs were viewed as part of their core practice. They felt that it would take several years. In Organisation A, the Implementation Lead felt that PROMs were not necessarily compatible with their community-led organisational culture and thus was less inclined to make changes.

At this stage it’s about creating a culture and creating infrastructure within the organisation. [Implementation Lead B]

#### Having a trial period

Both TSOs had a trial period, where they tested their bespoke measures on service-users to ensure they were acceptable. In Organisation B, this was a formalised process, they piloted the measure over a few months with service-users from different wellbeing services. In Organisation A, they tried the measure in one group activity, on one occasion. Interviewees from both organisations felt having a trial was useful as reassured front-line workers that service-users felt the measure was appropriate.

Email exchange with [Name of front-line worker] about how the trialling period went. They had used it in the [name of wellbeing activity]. People had found it easy and straightforward. [Name of front-line workers] feels it would be suitable to use with service-users. [Extract from reflective diary]

#### Challenges sustaining the use of PROMs with front-line workers needing ongoing support

**Fig. 2:**
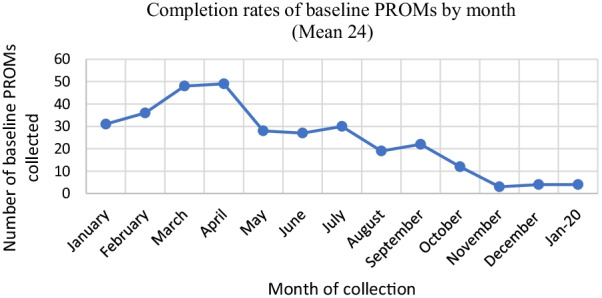
Monthly baseline completion rates in Organisation B between January 2019 and January 2020

Despite the organisations designing their own measure that was acceptable to them and investing time in implementation, the quantitative data indicated issues with sustaining the use of PROMs. In Organisation B, rates of collecting follow-up measures as a proportion of baseline measures per front-line worker ranged between 8.8 and 41.9% (Mean: 19.8%). Furthermore, the collection of PROMs decreased over time (Fig. 2). This was surprising as the expectation was that the use of measures would increase as staff became more experienced at collecting PROMs. Some front-line workers discussed that whilst the bespoke measure was an improvement on previous measures, they still believed that using PROMs was not compatible with the relationships they had built with service-users. Other front-line workers reported forgetting to administer the measures. Reflecting on this, Implementation Lead B felt that front-line workers needed greater ongoing support to use PROMs. For example, reminders within supervision to iterate that collecting PROMs was part of people’s job.

Just to see, it is still here, this is the usage, we need to keep on using it, and just to encourage the senior workers to embed that as part of their supervision, cos I actually put it on the supervision form. [Implementation Lead B]

## Discussion

The TSOs invested significant time and resources into implementation which included designing their own measures. Despite aspirations to utilise their choice of measure, the TSOs had to prioritise PROMs mandated by commissioners. Furthermore, beliefs, culture and conflicting priorities interacted to create significant barriers to collecting measures, raising questions about the feasibility of using PROMs in some organisations. Despite the increasing evidence-base on best practice when implementing PROMs, the organisations struggled to address barriers in the short time frame of this study.

### Strengths and limitations

The study had three strengths. (1) Through being involved with the organisations for over a year, we were able to observe the interplay of different facilitators and barriers. (2) Taking a mixed methods approach enabled us to observe how PROMs collection rates reduced and explore reasons for this. (3) Utilising CBPR ensured a reciprocal approach, with the TSOs receiving support from the researchers. There were four limitations. (1) The study would have benefited from the TSOs being more involved in the evaluation, for example the Implementation Leads keeping reflective diaries. (2) We were not able to undertake quantitative analysis of collected PROMs in Organisation A because they had not collected a sufficient number of measures. However, this was a finding in itself. (3) Sometimes organisations made decisions which did not align with the PROMs’ implementation evidence-base and the researchers could have been more persuasive whilst still adhering to participatory principles. (4) Implementation took longer than planned and thus the study finished whilst the organisations were still on their implementation journey. Consequently, we were unable to explore the organisations’ experiences later on in their implementation journey. For example, it would have been valuable to have re-interviewed senior managers about the decreasing PROMs completion rates.

### Comparison with existing literature

We compared our findings with a range of existing studies on implementing PROMs. We build upon previous research by identifying how using an appropriate measure may be less of an influencing factor than external pressures, input from Implementation Leads and the intersection of organisational priorities and culture. Whilst our research focused specifically on TSOs, our findings reflect factors identified in other settings.

Our finding that external commissioners had considerable influence over the use of PROMs because of TSO’s receiving short-term funding reflects experiences in both primary care [44] and mental health services [45]. Both TSOs chose to design a bespoke measure rather than use a validated one, reflecting concerns raised by others that there are issues with using existing validated PROMs within the third sector. Foster et al. (2020) found that the use of bespoke measures was common practice within the sector [46], which raises concerns about the psychometric validity of the measures being used. Researchers have proposed developing a specific third sector PROM that is psychometrically tested [9]. However an item bank format, which can be adapted to the specific context may be more acceptable [47].

It appeared that using an acceptable measure was not the only barrier to overcome and that other challenges were more influential. Previous studies have focused on whether an organisation’s culture is amenable to PROMs [14, 48]. Our research identified that organisational culture is not static, if leaders invest time and resources, the culture can evolve to become more outcomes-focused. However, Implementation Leads and managers need to buy into the concept of outcome measurement to enable this. We identified that implementation takes a considerable period of time, which reflects a previous study where it took three years to implement PROMs [49]. In our study, the use of PROMs decreased over time, indicating that ongoing support is needed to sustain their use, which reflects other studies [18]. Our experience indicates that TSOs and commissioners may want to explore alternative methods to PROMs [22, 23].

### Implications

We found many similar facilitators and barriers to other studies, indicating that organisations can learn from each other irrespective of their specific focus. Similarities included the importance of having buy in from stakeholders, the influence of organisational culture, the need for ongoing training and the amount of time and resources implementation takes. Organisations may not have full control over their PROMs processes because they must respond to requirements from commissioners. Consequently, organisations may have to implement different PROMs and change their processes to meet external needs, making implementation an ongoing rather than finite process. Developing a third sector specific measure which takes an ‘item bank’ format may overcome the challenges of both acceptability and psychometric validity. Organisations need to be prepared to invest considerable time (often years) and resources into progressing implementation of PROMs including making outcome measurement part of the culture and providing ongoing support to sustain their use. However, even with these changes it is possible that organisations may not have the resources to address some implementation barriers such as Implementation Leads having competing priorities. A solution may be to look for alternatives to PROMs.

### Conclusion

Despite investing considerable time and resources into implementation, and using a bespoke PROM that staff valued, two TSOs struggled to implement measures within their practice, partly because not all stakeholders valued PROMs. This raises questions about whether the issue with PROMs is less about specific measures and more about the feasibility of using them in this context.

## Supplementary Information

**Additional file 1**. Mixed Methods Appraisal Tool

**Additional file 2**. Organisation B’s outcome measure

**Additional file 3**. Organisation A’s outcome measure

## Data Availability

Due to the nature of the study, the data and material relating to the study is not publicly available.

## Abbreviations

AF: Initials of Lead Researcher (Alexis Foster-Lead Author)
ASCOT: Adult Social Care Outcomes Toolkit
CBPR: Community-based Participatory Research
CFIR: Consolidated Framework for Implementation Research
JH: Initial of Janet Harris, participatory research expert and co-author
ONS-4: Office for National Statistics 4
PROMs: Patient Reported Outcome Measures
PROMIS: Patient-reported Outcomes Measurement Information System
SPSS: Statistical Package for the Social Sciences
SWEMWBS: Short Warwick-Edinburgh Mental Wellbeing Scale
TSOs: Third Sector Organisations
WEMWBS: Warwick-Edinburgh Mental Wellbeing Scale
