# Supplementary material for: Using co-production to implement patient reported outcome measures in third sector organisations: a mixed methods study
Source: J Patient Rep Outcomes. 2022 Jul 19;6:78. doi: 10.1186/s41687-022-00485-4 (PMC9296723; doi:10.1186/s41687-022-00485-4)
Supplement: Supplementary file 1 — Additional file 1. Mixed Methods Appraisal Tool [file 41687_2022_485_MOESM1_ESM.docx]

**Supplementary file 1- Mixed Methods Appraisal Tool**

| **Category of study designs** | **Methodological quality criteria** | **Responses** | | | |
| --- | --- | --- | --- | --- | --- |
|  |  | **Yes** | **No** | **Can’t tell** | **Comments** |
| **Screening questions (for all types)** | S1. Are there clear research questions? | X |  |  |  |
|  | S2. Do the collected data allow to address the research questions? | X |  |  |  |
|  | *Further appraisal may not be feasible or appropriate when the answer is ‘No’ or ‘Can’t tell’ to one or both screening questions.* |  |  |  |  |
| **1. Qualitative** | 1.1. Is the qualitative approach appropriate to answer the research question? | X |  |  |  |
|  | 1.2. Are the qualitative data collection methods adequate to address the research question? | X |  |  |  |
|  | 1.3. Are the findings adequately derived from the data? | X |  |  |  |
|  | 1.4. Is the interpretation of results sufficiently substantiated by data? | X |  |  |  |
|  | 1.5. Is there coherence between qualitative data sources, collection, analysis and interpretation? | X |  |  |  |
| **2. Quantitative randomised controlled trials** | 2.1. Is randomisation appropriately performed? | N/A |  |  |  |
|  | 2.2. Are the groups comparable at baseline? | N/A |  |  |  |
|  | 2.3. Are there complete outcome data? | N/A |  |  |  |
|  | 2.4. Are outcome assessors blinded to the intervention provided? | N/A |  |  |  |
|  | 2.5 Did the participants adhere to the assigned intervention? | N/A |  |  |  |
| **Category of study designs** | **Methodological quality criteria** | **Responses** |  |  |  |
|  |  | **Yes** | **No** | **Can’t tell** | **Comments** |
| **3. Quantitative nonrandomized** | 3.1. Are the participants representative of the target population? | N/A |  |  |  |
|  | 3.2. Are measurements appropriate regarding both the outcome and intervention (or exposure)? | N/A |  |  |  |
|  | 3.3. Are there complete outcome data? | N/A |  |  |  |
|  | 3.4. Are the confounders accounted for in the design and analysis? | N/A |  |  |  |
|  | 3.5. During the study period, is the intervention administered (or exposure occurred) as intended? | N/A |  |  |  |
| **4. Quantitative descriptive** | 4.1. Is the sampling strategy relevant to address the research question? | X |  |  |  |
|  | 4.2. Is the sample representative of the target population? |  |  | X |  |
|  | 4.3. Are the measurements appropriate? | X |  |  |  |
|  | 4.4. Is the risk of nonresponse bias low? |  | X |  |  |
|  | 4.5. Is the statistical analysis appropriate to answer the research question? | X |  |  |  |
| **5. Mixed methods** | 5.1. Is there an adequate rationale for using a mixed methods design to address the research question? | X |  |  |  |
|  | 5.2. Are the different components of the study effectively integrated to answer the research question? | X |  |  |  |
|  | 5.3. Are the outputs of the integration of qualitative and quantitative components adequately interpreted? | X |  |  |  |
|  | 5.4. Are divergences and inconsistencies between quantitative and qualitative results adequately addressed? | X |  |  |  |
|  | 5.5. Do the different components of the study adhere to the quality criteria of each tradition of the methods involved? | X |  |  |  |
