# Supplementary figures and images for: Using co-production to implement patient reported outcome measures in third sector organisations: a mixed methods study
Source: J Patient Rep Outcomes. 2022 Jul 19;6:78. doi: 10.1186/s41687-022-00485-4 (PMC9296723; doi:10.1186/s41687-022-00485-4)

## Supplementary file 2- Organisation B’s PROM


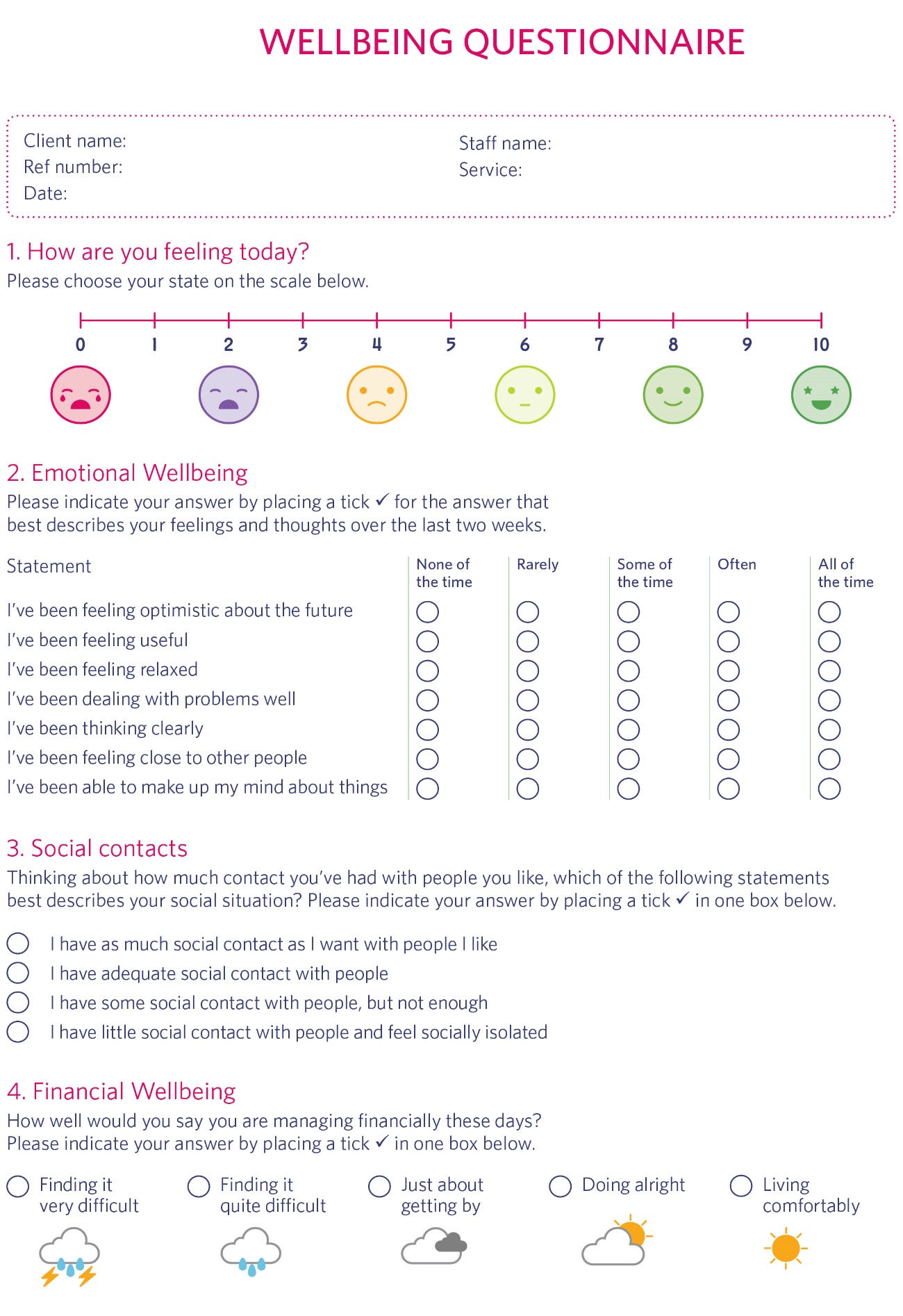

Supplement: Supplementary file 2 — Additional file 2. Organisation B’s outcome measure [file 41687_2022_485_MOESM2_ESM.docx]

## Supplementary file 3- Organisation A’s PROM


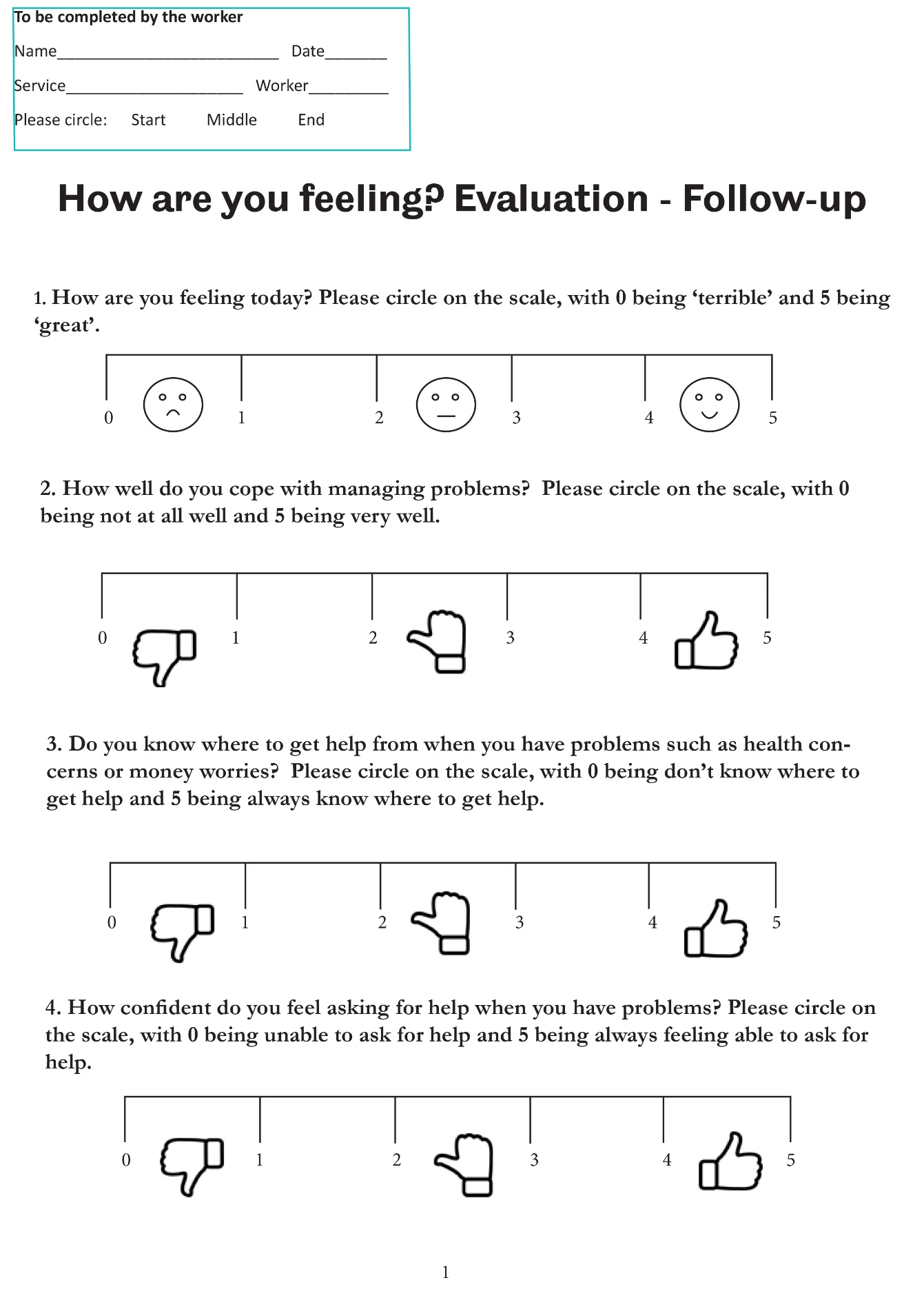


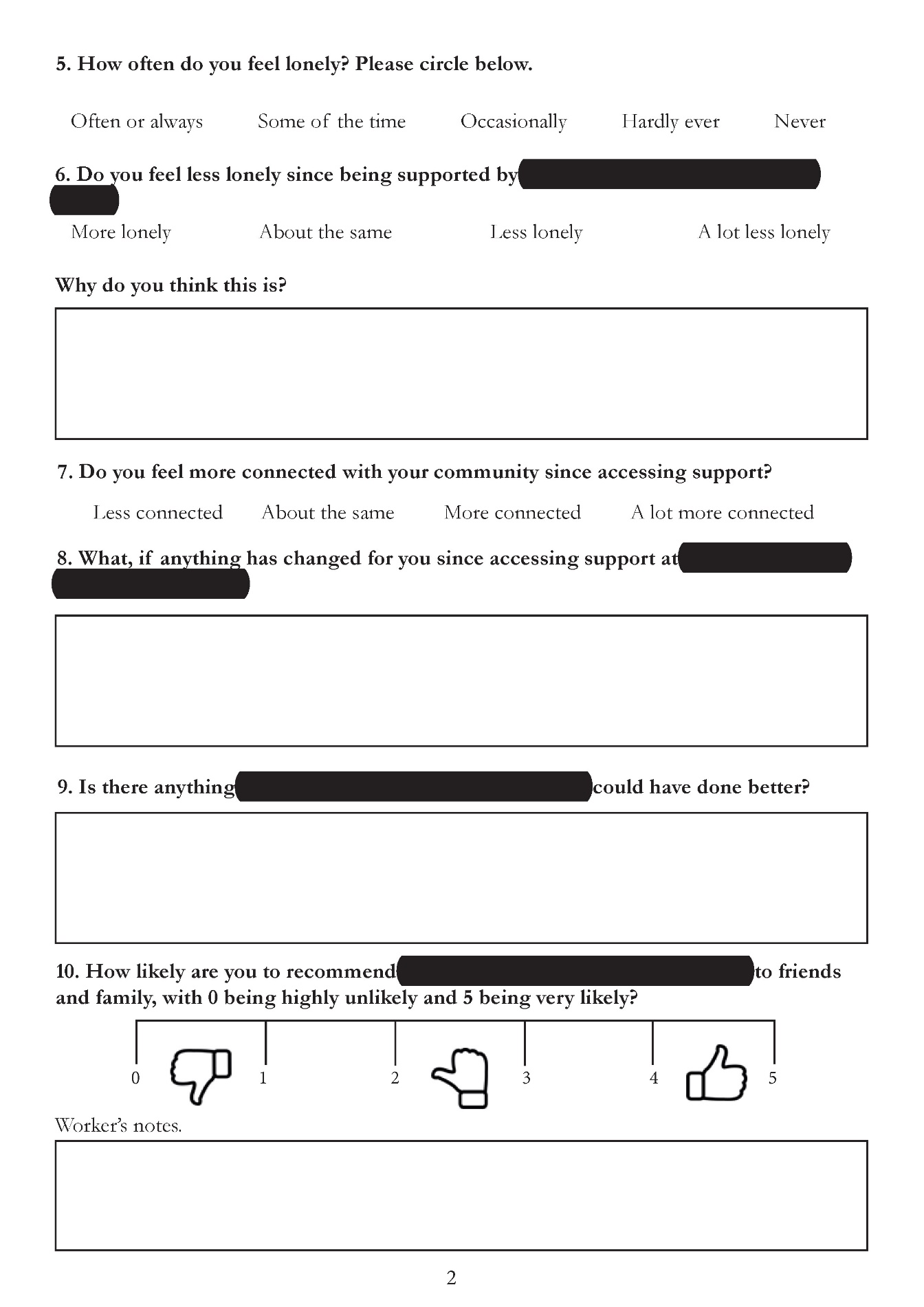

Supplement: Supplementary file 3 — Additional file 3. Organisation A’s outcome measure [file 41687_2022_485_MOESM3_ESM.docx]
